# E3 Ubiquitin Ligase Midline 1 Regulates Endothelial Cell ICAM-1 Expression and Neutrophil Adhesion in Abdominal Sepsis

**DOI:** 10.3390/ijms24010705

**Published:** 2022-12-31

**Authors:** Feifei Du, Avin Hawez, Zhiyi Ding, Yongzhi Wang, Carl-Fredrik Rönnow, Milladur Rahman, Henrik Thorlacius

**Affiliations:** Department of Clinical Sciences, Malmö, Section for Surgery, Lund University, 214 28 Malmö, Sweden

**Keywords:** adhesion, sepsis, lung, Midline 1, endothelial cells

## Abstract

Septic lung damage is associated with endothelial cell and neutrophil activation. This study examines the role of the E3 ubiquitin ligase midline 1 (Mid1) in abdominal sepsis. Mid1 expression was increased in endothelial cells derived from post-capillary venules in septic mice and TNF-α challenge increased Mid1 levels in endothelial cells in vitro. The siRNA-mediated knockdown of Mid1 decreased TNF-α-induced upregulation of ICAM-1 and neutrophil adhesion to endothelial cells. Moreover, Mid1 silencing reduced leukocyte adhesion in post-capillary venules in septic lungs in vivo. The silencing of Mid1 not only decreased Mid1 expression but also attenuated expression of ICAM-1 in lungs from septic mice. Lastly, TNF-α stimulation decreased PP2Ac levels in endothelial cells in vitro, which was reversed in endothelial cells pretreated with siRNA directed against Mid1. Thus, our novel data show that Mid1 is an important regulator of ICAM-1 expression and neutrophil adhesion in vitro and septic lung injury in vivo. A possible target of Mid1 is PP2Ac in endothelial cells. Targeting the Mid1-PP2Ac axis may be a useful way to reduce pathological lung inflammation in abdominal sepsis.

## 1. Introduction

Abdominal sepsis is associated with morbidity and mortality. Pulmonary damage is the most critical feature in sepsis and numerous studies have shown that neutrophil recruitment is a rate-limiting step in septic lung injury [1,2]. Pulmonary accumulation of neutrophils is a multistep process, including rolling, firm adhesion and extravasation [3,4]. Neutrophil–endothelial cell interactions are mediated by specific adhesion molecules. Neutrophil rolling is supported by the selectin family of adhesion molecules [5,6,7]. Firm adhesion of neutrophils to microvessels is mediated by β2-integrins expressed on neutrophils binding to ICAM-1 on endothelial cells [8,9]. ICAM-1 is constitutively expressed at low levels on endothelial cells but is rapidly increased after stimulation with pro-inflammatory compounds, such as TNF-α [10,11]. Targeting ICAM-1 in experimental models of lung inflammation reduces leukocyte infiltration and tissue damage [12,13]. Although ICAM-1 appears to be important in the etiology of sepsis, the regulatory mechanisms of ICAM-1 expression in sepsis is not well known.

Midline 1 (Mid1), a microtubule-associated E3 ubiquitin ligase (also known as tripartite motif-containing protein 18, TRIM18) is known to be involved in organ development and diseases, such as cancer, dementia and allergic inflammation [14,15,16,17]. A recent study reported that Mid1 can regulate pro-inflammatory transcription factors, including NF-kB, p38 MAPK and JNK [18]. These transcription factors are known to control pro-inflammatory signaling pathway in endothelial cells [19,20,21,22]. The target protein of Mid1 is protein phosphatase 2A (PP2A) complex and levels of PP2A has been shown to regulate cancer cell proliferation, migration, cytoskeletal rearrangement and apoptosis [23]. The PP2A complex has three subunits, scaffolding subunit A, regulatory subunit B and catalytic subunit C (PP2Ac), of which catalytic subunit PP2Ac interacts with Mid1 protein [24]. Mid1 is known to decrease protein phosphatase 2A (PP2A) activity by ubiquitin-specific degradation of its catalytic subunit PP2Ac. For instance, a previous study showed that silencing of Mid1 by siRNA reverses degradation of PP2Ac and increases activity of PP2A in a model of allergic airway inflammation [18]. However, the role of Mid1 in regulating endothelial cell expression of ICAM-1 and neutrophil adhesion during sepsis has not been investigated.

Based on the considerations above, the aim of this study was to determine the role of E3 ubiquitin ligase Mid1 in endothelial cell activation, ICAM-1 expression and neutrophil adhesion in abdominal sepsis.

## 2. Results

### 2.1. Mid1 Expression in Lung Post-Capillary Venules of Cecal Ligation Puncture (CLP) Mice and Mouse Endothelial Cell Line

**Figure 1:**
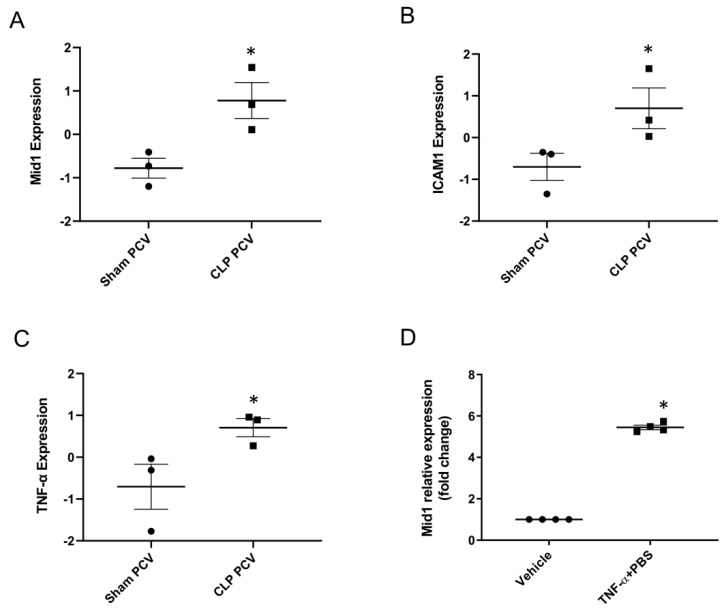
Mid1 expression in endothelial cell lines and lung post-capillary venules of septic mice. (**A**) Mid1, (**B**) ICAM-1, (**C**) TNF-α expression in the lung post-capillary venules. Expression was analyzed in the RNAseq data using R program (DESeq2). Samples were collected 4 h after induction of abdominal sepsis, and sham mice served as negative control, *n* = 3. (**D**) eEnd.2 cells were treated with vehicle and TNF-α (100 ng/mL) for 1 h, and then the expression of Mid1 mRNA was determined by RT-qPCR. Data are mean ± SEM and *n* = 4. * *p* < 0.05 vs. Sham PCV or Vehicle.

To study Mid1 gene expression in lung endothelium of experimental abdominal sepsis, RNAseq data (GSE169532) of post-capillary venules were analyzed by R program. It was observed that Mid1 expression is significantly increased in the post-capillary venules of CLP animal than in the post-capillary venules of sham animals (Figure 1A). We also analyzed ICAM-1 and TNF-α expression in the RNAseq data and observed that CLP caused significant increase in ICAM-1 and TNF-α expression in the post-capillary venules compared to sham post-capillary venules (Figure 1B,C). We then determined expression levels of Mid1 in cultured endothelial cells by RT-qPCR 1h after stimulation with TNF-α. It was found that TNF-α challenge elevated Mid1 expression more than 5-fold compared with unstimulated samples (Figure 1D), indicating that endothelial cell expression of Mid1 is increased in response to both sepsis and TNF-α exposure.

### 2.2. Mid1 Silencing Reduces ICAM-1 Expression and Neutrophil Adhesion In Vitro

**Figure 2:**
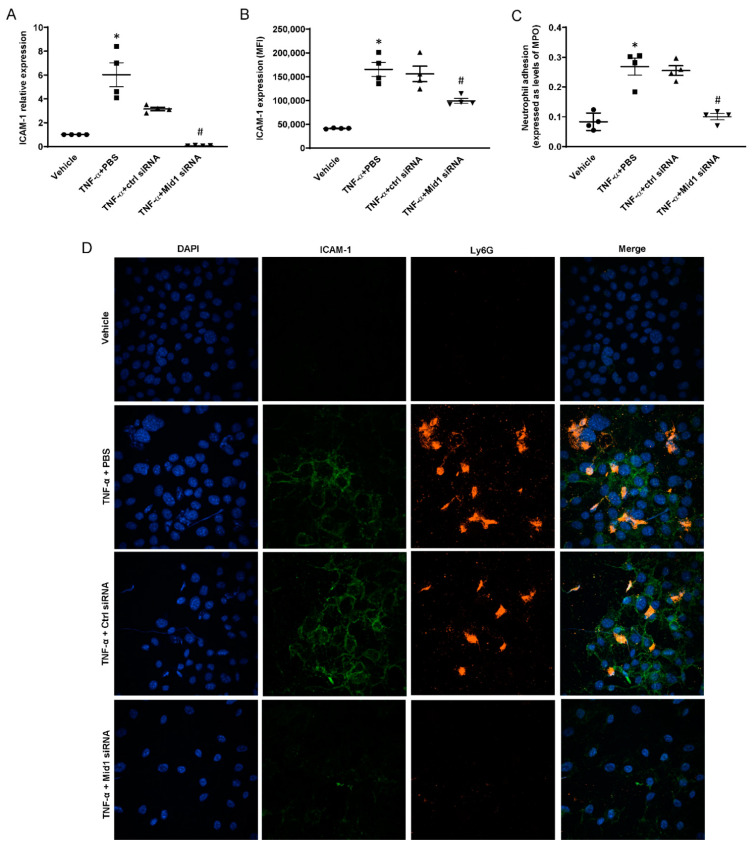
Mid1 siRNA reduced the expression of ICAM-1 and neutrophil adhesion in vitro. eEnd.2 cells were transfected with 100 nM negative control siRNA and Mid1 siRNA for 24 h, then stimulated with 100 ng/mL TNF-α. (**A**) ICAM-1 mRNA expression was determined by RT-qPCR 1 h after stimulation. (**B**) ICAM-1 surface expression was determined by flow cytometry 3 h after stimulation. For the assay of neutrophil adhesion to endothelial cells, eEnd.2 cells were treated as described above, and freshly isolated neutrophils were added to each well for 1 h. (**C**) Neutrophil adhesion was examined in terms of MPO levels, (**D**) ICAM-1 expression and neutrophil adhesion were examined by confocal microscopy. Data are mean ± SEM and *n* = 4. * *p* < 0.05 vs. Vehicle, # *p* < 0.05 vs. TNF-α + Control siRNA.

We next examined whether Mid1 regulates ICAM-1 expression and neutrophil adhesion on TNF-α-activated endothelial cells by RT-qPCR and flow cytometry. It was observed that challenge with TNF-α increased ICAM-1 mRNA expression more than 6-fold and ICAM-1 surface expression more than 4-fold in endothelial cells compared with unstimulated cells (Figure 2A,B). Endothelial cell transfection with 100 nM Mid1 siRNA decreased TNF-α-induced ICAM-1 expression by 98% at RNA levels compared with negative control siRNA group (Figure 2A). Flow Cytometry analysis showed that endothelial cell transfection with Mid1 siRNA significantly reduced TNF-α-provoked ICAM-1 surface expression compared to negative control siRNA group (Figure 2B), suggesting that Mid1 regulates TNF-α-dependent ICAM-1 expression in endothelial cells. Since ICAM-1 is known to be involved in firm adhesion of leukocytes on endothelium, we then performed an in vitro neutrophil adhesion assay on stimulated endothelial cells. Cells were transfected and stimulated with TNF-α, then freshly isolated neutrophils were added to each well as described in the Materials and Methods section. After careful washing, neutrophil adhesion on activated endothelial cells was calculated in terms of MPO levels (ng/mL) in each well. It was found that MPO levels increased significantly in the TNF-α-stimulated group. Endothelial cell transfection with Mid1 siRNA attenuated MPO levels by 60% compared to negative control siRNA group (Figure 2C). In addition, we analyzed neutrophil adhesion and ICAM-1 expression on stimulated endothelial cells by use of confocal microscopy. It was found that TNF-α stimulation increased ICAM-1 expression and neutrophil adhesion to activated endothelial cells. Notably, endothelial cell transfection with Mid1 siRNA reduced ICAM-1 expression and the number of adherent neutrophils (Figure 2D), suggesting that neutrophil adhesion to endothelial cells is regulated by Mid1-dependet expression of ICAM-1. Furthermore, we analyzed VCAM-1 expression on lung post-capillary venules and observed that CLP caused a significant increase in VCAM-1 expression in the post-capillary venules compared to sham post-capillary venules (Supplementary Figure S1A). Next, we evaluated the role of Mid1 on VCAM-1 expression and found that endothelial cell transfection with Mid1 siRNA, decreased TNF-α-induced VCAM-1 expression by 52% at mRNA levels (Supplementary Figure S1B) and by 51% on the surface of endothelial cells (Supplementary Figure S1C) compared with negative control siRNA group, suggesting that Mid1 also regulates TNF-α-dependent VCAM-1 expression in endothelial cells.

### 2.3. Mid1 Silencing Reduces Leukocyte Adhesion In Vivo

**Figure 3:**
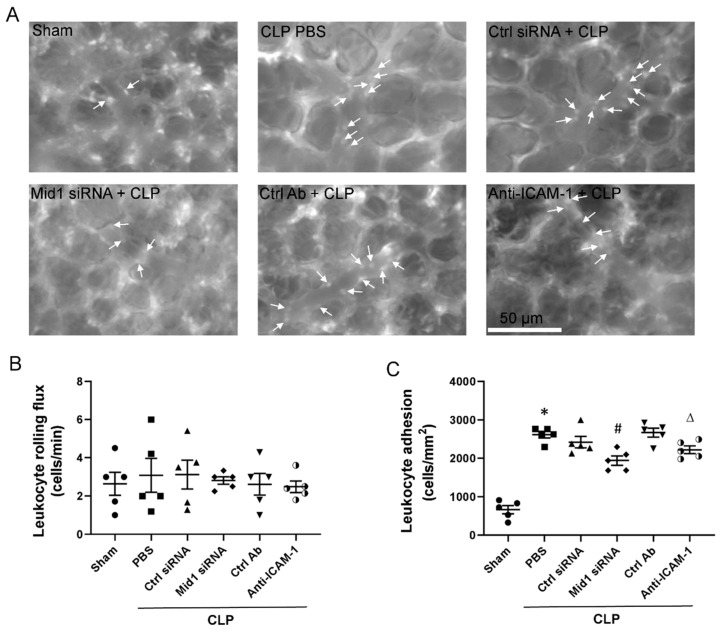
Leukocyte recruitment in vivo. (**A**) Representative images showing leukocytes adhesion to lung post-capillary venules. Scale bars = 50 µm. Leukocyte (**B**) rolling flux and (**C**) adhesion in mouse lung tissues 4 h after CLP induction; for sham group, animals underwent sham procedure as described in method. Animals were pretreated with negative control siRNA, Mid1 siRNA, anti-ICAM-1 and an isotype matched control antibody as described in the Materials and Methods section. Control animals were injected with PBS. Data are mean ± SEM and *n* = 5. * *p* < 0.05 vs. Sham, # *p* < 0.05 vs. Control siRNA + CLP, ∆ *p* < 0.05 vs. Control antibody + CLP.

To explore the role of Mid1 in leukocyte adhesion in septic mice, we injected Mid1 siRNA intravenously into mice before CLP and examined leukocyte–endothelium interactions in post-capillary venules by use of intravital microscopy. CLP triggered a clear-cut increase in the number of firmly adherent leukocytes in post-capillary venules (Figure 3A,C). Notably, we observed that silencing of Mid1 significantly reduced firm leukocyte adhesion in post-capillary venules (Figure 3A,C). Moreover, immunoneutralization of ICAM-1 significantly attenuated CLP-induced leukocyte adhesion in post-capillary venules in the lung (Figure 3A,C). In contrast, it was found that leukocyte rolling flux was not significantly changed after induction of CLP (Figure 3B).

### 2.4. Mid1 Silencing Reduces ICAM-1 Expression In Vivo

**Figure 4:**
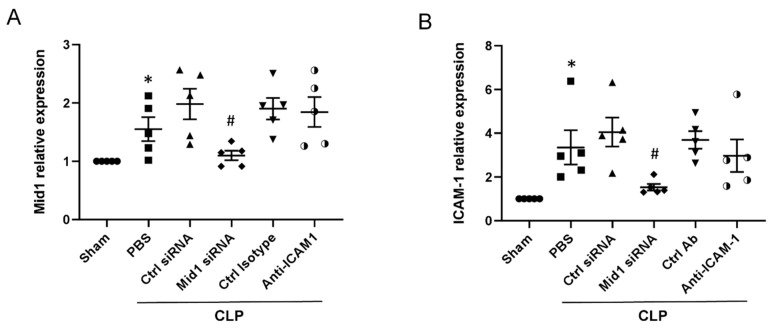
ICAM-1 mRNA expression in lung tissue. Lung tissues were collected, and mRNA was isolated to determine the expression of (**A**) Mid1 and (**B**) ICAM-1. Animals were pretreated with negative control siRNA, Mid1 siRNA, anti-ICAM-1 and an isotype matched control antibody as described in the Materials and Methods section. Control animals were injected with PBS. Data are mean ± SEM and *n* = 5. * *p* < 0.05 vs. Sham, # *p* < 0.05 vs. Control siRNA + CLP.

We then examined Mid1 expression and ICAM-1 expression in lung tissues of mice. Mid1 expression in lung tissues increased after CLP induction, while administration of Mid1 siRNA reduced the Mid1 expression in lung tissues of CLP mice compared to negative control siRNA-treated group (Figure 4A). Furthermore, ICAM-1 expression in lung tissues of CLP mice increased more than 3-fold compared to sham group, and Mid1 silencing reduced pulmonary expression of ICAM-1 by more than 60% compared to negative control siRNA-treated group. Notably, the injection of anti-ICAM-1 antibody had no impact on the expression of Mid1 and ICAM-1 in lung tissues of CLP mice (Figure 4A,B).

### 2.5. Mid1 Regulates PP2Ac Expression

**Figure 5:**
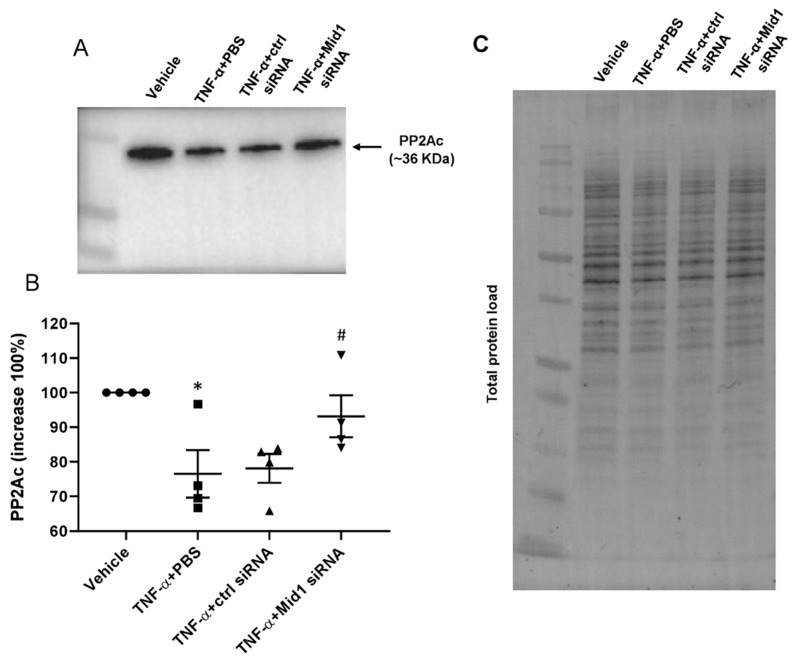
Mid1 regulates PP2Ac expression. eEnd.2 cells were transfected with 100 nM negative control siRNA or Mid1 siRNA for 24 h, then stimulated with 100 ng/mL TNF-α for 1 h. (**A**) PP2Ac expression was examined by Western blot. (**B**) Increase percentage of PP2Ac after normalization with total protein load in each well. (**C**) Total protein load used to normalize PP2Ac bands. Data are mean ± SEM and *n* = 4. * *p* < 0.05 vs. Vehicle, # *p* < 0.05 vs. TNF-α + Control siRNA.

To further explore the potential mechanism of the Mid1-mediated target protein degradation, we examined the levels of PP2Ac protein in transfected cells. eEnd.2 cells were transfected with 100 nM Mid1 siRNA or control siRNA for 24 h and then stimulated with 100 ng/mL TNF-α for 1 h, the levels of PP2Ac were evaluated by Western blotting. Levels of PP2Ac were calculated after normalizing the respective band with total protein load of each lane (Figure 5A,C). It was observed that stimulation with TNF-α significantly reduced PP2Ac protein levels (Figure 5A,B). In contrast, the levels of PP2Ac were increased in Mid1 siRNA transfected cells compared to negative control siRNA transfected cells (Figure 5A,B), suggesting that Mid1 plays a role in endothelial cells activation through regulating PP2Ac levels.

### 2.6. Mid1 Regulates ICAM-1 Expression in Human Lung Microvascular Endothelial Cells (HLMVEC) In Vitro

**Figure 6:**
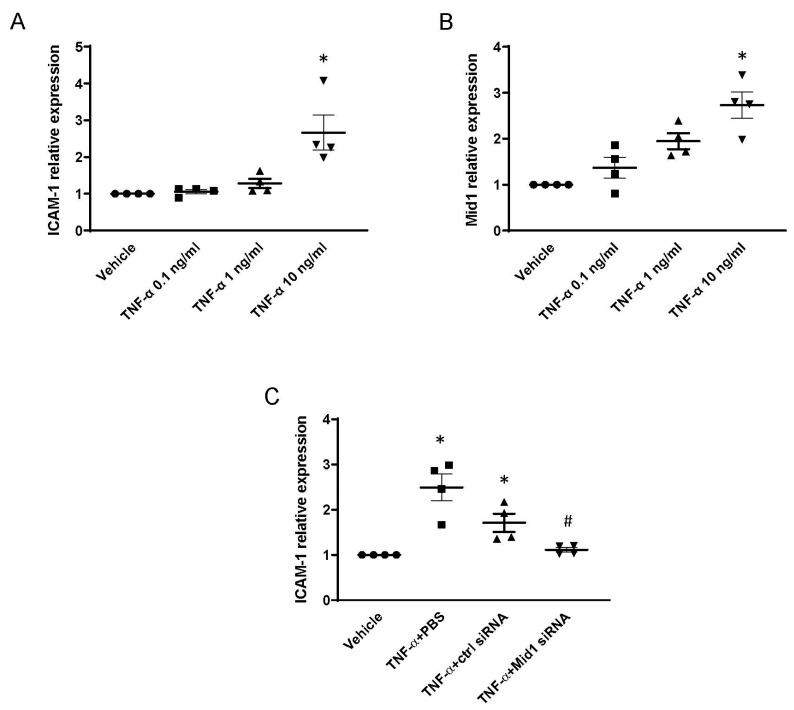
TNF-α increased ICAM-1 expression in primary HLMVEC and silencing of Mid1 by siRNA reduced the expression of ICAM-1 in vitro. HLMVEC were treated with vehicle and TNF-α (0.1–10 ng/mL) for 1 h and then expression of (**A**) ICAM-1 and (**B**) Mid1 mRNA was determined by RT-qPCR. (**C**) HLMVEC were transfected with 100 nM negative control siRNA and Mid1 siRNA for 24 h followed by stimulation with 10 ng/mL human TNF-α. ICAM-1 mRNA expression was determined by RT-qPCR 1 h after TNF-α stimulation. Data are mean ± SEM and *n* = 4. * *p* < 0.05 vs. Vehicle, # *p* < 0.05 vs. TNF-α + Control siRNA.

We next examined whether Mid1 regulates ICAM-1 expression in adult primary human pulmonary microvascular endothelial cells. HLMVEC were stimulated with recombinant human TNF-α (0.1–10 ng/mL) for 1 h, and ICAM-1 and Mid1 expression were examined by RT-qPCR as described above. It was observed that challenge with 10 ng/mL of TNF-α increased ICAM-1 and Mid1 mRNA expression in HLMVEC compared with unstimulated cells (Figure 6A,B). Next, we transfected HLMVEC with 100 nM human Mid1 siRNA. Transfection with Mid1 siRNA decreased TNF-α-induced mRNA expression of ICAM-1 expression by 84% compared with negative control siRNA group (Figure 6C), suggesting that Mid1 also regulates TNF-α-dependent ICAM-1 expression in primary human microvascular endothelial cells.

## 3. Discussion

Management of patients with abdominal sepsis is challenging and limited to antibiotics and supportive care. New and effective targets are needed in order to improve treatment of patients with septic lung damage. This study demonstrates that Mid1 is a novel regulator of endothelial cell activation and inflammation. Thus, we found that Mid1 regulates endothelial cell expression of ICAM-1 and neutrophil adhesion in vitro and in vivo, indicating that Mid1 could be a useful target to limit pathological inflammation in abdominal sepsis.

Leukocyte recruitment in the lung microvasculature is a complex process. It has been shown that mechanical trapping of stiff leukocytes is a prominent feature in lung capillaries, whereas active leukocyte-endothelium interactions, including rolling and firm adhesion, are relatively more important in post-capillary venules [25,26]. Thus, new targets for inhibiting leukocyte–endothelium interactions in the inflamed lung should focus on molecular aspects of endothelial cells derived from post-capillary venules. Based on transcriptomic analysis of isolated microvascular endothelial cell subsets, we found that post-capillary venular endothelial from CLP mice expressed significantly higher levels of Mid1 compared to sham mice. This finding extends on a previous study reporting that allergen exposure to sensitized mice causes Mid1 upregulation in bronchial epithelial cells [18]. Together, these observations suggest that Mid1 could play multiple roles in diverse lung-derived cells in pulmonary inflammation. In addition, we observed that TNF-α challenge increased Mid1 expression in endothelial cells in vitro, suggesting that Mid1 could be a common feature in endothelial cells during inflammatory reactions. Knowing that the PP2A complex positively regulates several pro-inflammatory pathways, including NF-kappa B, p38 MAPK and JNK [18,27,28], and is the main target of Mid1 in epithelial lung adenocarcinoma, bronchial epithelium in asthma and primary cerebellar granule neurons [18,29,30], it was of great interest to study levels of PP2Ac in endothelial cells. Herein, we observed that TNF-α stimulation decreased endothelial cell expression of PP2Ac. Interestingly, silencing of Mid1 reversed the TNF-α-induced reduction in PP2Ac. These findings add endothelial cells to the line of cells, including cancer cells and neural cells, in which Mid1 regulates PP2Ac expression. Several studies have shown that TNF-α-induced ICAM-1 expression is regulated by NF kappa B signaling [31,32,33]. Based on previous studies and our observation it could be suggested that PP2Ac might regulate ICAM-1 expression in endothelial cells via NF kappa B signaling pathway. Further studies are required to explore signaling mechanisms involved in endothelial ICAM-1 expression.

Specific adhesion molecules regulate dynamic leukocyte–endothelium interactions [34]. For example, endothelial cell expression of P- and E-selectin support a rolling adhesive interaction slowing down circulating leukocytes and allow them to detect chemokines on the surface of the microvascular wall [6]. Moreover, firm adhesion of leukocytes to endothelial cells is mediated by integrin receptors, including ICAM-1 and VCAM-1 [35,36,37]. In the present study, we found that stimulation with TNF-α markedly increased ICAM-1 expression in both murine and human endothelial cells, which is in line with previous studies. To assess the role of Mid1 in TNF-α-induced upregulation of ICAM-1, we reduced Mid1 expression by siRNA in endothelial cells before the challenge with TNF-α. Mid1 silencing markedly decreased ICAM-1 expression in endothelial cells exposed to TNF-α. Moreover, we also observed that silencing of Mid1 in endothelial cells reduced TNF-α-provoked firm adhesion of neutrophils. We next studied dynamic leukocyte–endothelium interactions on the pulmonary post-capillary venules in septic mice. It was found that CLP caused no significant change in leukocyte rolling, but instead, a significant increase was found in firm leukocyte adhesion, as reported in previous investigations [38,39]. Notably, in vivo administration of Mid1 siRNA significantly decreased CLP-induced leukocyte adhesion in pulmonary post-capillary venules, suggesting that Mid1 also regulates leukocyte adhesion in vivo. Moreover, immunoneutralization of ICAM-1 attenuated leukocyte adhesion supporting the notion that ICAM-1 mediates adhesive interactions between leukocytes and endothelial cells in vivo [36,37]. In addition, our results show that Mid1 silencing decreased both Mid1 and ICAM-1 in the lungs of septic mice. These results indicate that Mid1 plays a functional role in regulating ICAM-1-depedent leukocyte adhesion in septic lung injury.

Taken together, our novel findings suggest that Mid1 is an important regulator of ICAM-1 expression and neutrophil adhesion in vitro and septic lung injury in vivo. A possible target of Mid1 is PP2Ac in endothelial cells. Thus, targeting the Mid1-PP2Ac axis may be a useful way to reduce pathological lung inflammation in abdominal sepsis.

## 4. Materials and Methods

### 4.1. Animals

All experiments complied with the legislation on the protection of animals and were approved by the Regional Ethical Committee for Animal Experimentation at Lund University, Sweden (Permit number: 5.8.18-08769/2019). Male C57BL/6 mice (Janvier Labs, Le Genest-Saint-Isle, France) weighing 20–25 g were maintained in a pathogen-free facility on 12 h dark/light cycles and had free access to food and water. Mice were kept at a maximum of 5 mice per cage with environmental enrichment. Animals were anaesthetized by intraperitoneal administration of 75 mg/kg ketamine hydrochloride (Hoffmann-La Roche, Basel, Switzerland) and 25 mg/kg xylazine (Janssen Pharmaceutical, Beerse, Belgium). Animals were randomly assigned to experiments and 0.5 mg/kg buprenorphine hydrochloride (Schering-Plough, Berkeley Heights, NJ, USA) was given subcutaneously to relieve pain.

### 4.2. Experimental Protocol of Sepsis

Abdominal sepsis was induced in mice by cecal ligation puncture (CLP) procedure as previously described in detail [40]. Briefly, mice were anesthetized, and the cecum was exposed by a midline incision, milking stool backwards from the ascending colon, and cecum was ligated with a 5-0 silk suture. The cecum was kept moist with PBS (pH 7.4), and punctured twice by a 21-gauge needle on the antimesenteric border. Subsequently, the cecum was put back into the peritoneal cavity, and the abdominal incision was sutured. For sham group, the cecum was neither ligated nor punctured, but underwent identical laparotomy and resuscitation procedures. To study the role of Mid1, mice were intravenously injected with in vivo stable negative control siRNA and Mid1 siRNA (4 mg/kg, Accell siRNA Reagents, Dharmacon, Lafayette, CO, USA) 24 h and 2 h prior to CLP procedure. To examine the role of ICAM-1 in CLP-induced interaction between leukocytes and endothelial cells, the Isotype antibody (IgG2b) and anti-ICAM-1 antibody (YN1/1.7.4, BioXcell, Labombard Rd Lebanon, NH, USA) were applied intravenously 15 min before CLP induction. Animals were reanesthetized 4 h after CLP to perform lung intravital microscopy (IVM). Lung tissues were collected and homogenized for further examination.

### 4.3. Lung Intravital Fluorescence Microscopy

The stroke volume was adjusted transiently to 100 μL, and a right-sided pneumothorax was conducted by incising the right diaphragm. A parasternal thoracotomy was performed up to the level of the fourth intercostal space. Thus, the main part of the right thorax wall could be moved to the side. Great attention was paid not to manipulate the lung tissue directly, and the lung surface was kept moist by intermittently rinsing with saline (37 °C). A coverslip was fixed horizontally on the surface of right lung, Horizontal movements of the lung tissue could be minimized by modulating the positive end-expiratory pressure between 5 and 7 cm H_2_O and by adjusting the stroke volume (minimum: 150 μL) and frequency (minimum: 100 strokes/min) by use of a ventilator (Minivent type 845, Hugo Sachs Electronic-Harvard Apparatus, March-Hustetten, Germany). Fluorescence microscopy was performed after surgical preparation. Intravenous injection of 0.1 mL 0.1% rhodamine 6G (Sigma-Aldrich, St. Louis, MO, USA) for direct staining of leukocytes and 0.1 mL 5% FITC-dextran (MW 150,000; Sigma-Aldrich) for contrast enhancement of plasma. A modified Olympus microscope (BX50WI, Olympus Optical, Hamburg, Germany), equipped with a 100 W mercury lamp and filter sets for blue (450–490 nm excitation and >520 nm emission wavelength) and green (530–560 nm excitation; >580 nm emission) light epi-illumination was used to visualize the subpleural pulmonary microvasculature. By using a charge-coupled device video camera (FK 6990 Cohu, Pieper GmbH, Berlin, Germany), microscopic images of pulmonary post-capillary venules were televised and recorded digitally. For measurements, venules were randomly selected in each animal. To determine the leukocyte rolling, such cells were counted when passing a reference point in the venule per 20 s and expressed as cells per min. By counting the number of cells remaining stationary for 20 s in a 100 μm-long venule segment, firm adhesion was determined and expressed as cells/mm^2^.

### 4.4. RNA Sequence Data Analysis

We recently published data showing differential expression of lung post-capillary venules between 4 h Sham and CLP animals [41]. In that study, we FACS-sorted lung post-capillary venules as CD31+ICAM1+VCAM1+. Total RNA was extracted from sorted cells by RNeasy Plus Micro kit (Qiagen, West Sussex, UK) and RNA sequencing was conducted at the Center for Translational Genomics (CTG), Lund University and Clinical Genomics Lund, SciLifeLab. Differential expressions of Mid1, ICAM1 and TNF-α genes between Sham and CLP PCV were analyzed using DESeq2 in the statistical environment R (version 4.0.2).

### 4.5. Endothelial Cells Activation

The murine endothelioma cell line eEnd.2 (RRID:CVCL_6274) was cultured in DMEM containing 10% fetal calf serum, penicillin, and streptomycin as described previously (Williams et al., 1989). Thereafter, cells (2 × 10^5^/well) were planted into 6-well plates and incubated for 24 h. Cells were stimulated with 100 ng/mL murine TNF-α (PeproTech, Rocky Hill, NJ, USA) for 1 h when the confluence was about 90%. Primary human lung microvascular endothelial cells (HLMVEC) (Cell Applications. Inc., San Diego, CA, USA) were cultured in microvascular endothelium cell growth medium (Sigma-Aldrich) containing 10% fetal calf serum, penicillin, and streptomycin in flask coated with attachment factor solution (Sigma-Aldrich). Cells (1 × 10^5^/well) were planted into 6-well plates coated with attachment factor solution and incubated with 5% CO_2_, at 37 °C. Cells were stimulated with 0.1–10 ng/mL recombinant human TNF-α (Gibco, Carlsbad, CA, USA) for 1 h, when confluence was about 70–80%. HLMVEC with a total passage number of 8–9 were used for all experiments.

### 4.6. Transfection

eEnd.2 cells (2 × 10^5^/well) were seeded into 6-well plates for 24 h and transfected when the confluence was about 70% with silencer select negative control siRNA or Mid1 silencer select siRNA (Ambion, CA, USA) by use of TransIT-TKO transfection reagent (Mirus Bio, Madison, WI, USA) according to the manufacturer’s instructions. Then, 24 h after transfection, transfected cells were stimulated with 100 ng/mL murine TNF-α (PeproTech) for 1 h or 3 h, and then cells were collected for subsequent analysis. HLMVEC transfections were performed using human Mid1 silencer select siRNA (Ambion, CA, USA) according to the manufacturer’s instructions, and stimulation was performed with 10 ng/mL recombinant human TNF-α (Gibco). 

### 4.7. Quantitative Real-Time Polymerase Chain Reaction

Total RNA was isolated by use of Direct-Zol RNA MiniPrep kit (Zymo Research, Irvine, CA, USA) following the manufacturer’s instructions. Total RNA concentration was measured by Nanodrop spectrophotometer (Thermo Fisher Scientific, MA, USA) at 260 nm absorbance. Reverse transcription was performed by RevertAid First Strand cDNA Synthesis kit (Thermo Fisher Scientific) in a final volume of 20 μL according to the manufacturer’s instructions. Real-time PCR was conducted using SYBR Green dye (Takara Bio, Shiga, Japan) in a final volume of 25 μL. The primer sequences of mouse GAPDH, ICAM-1, Mid1 and VCAM-1 were as follows: GAPDH (forward) 5′-CATGTTCGTCATGGGGTGAACCA-3′, GAPDH (reverse) 5′-AGTGATGGCATGGACTGTGGTCAT-3′; ICAM-1 (forward) 5′-AGCACCTCCCCACCTACTTT-3′, ICAM-1 (reverse) 5′-AGCTTGCACGACCCTTCTAA-3′; Mid1 (forward) 5′-CACTCGCTGAAGGAAAATGACCA-3′, Mid1 (reverse) 5′-AATCCAAGGCAAAAGTGTCAAACG-3′; VCAM-1 (forward) 5′-AGTTGGGGATTCGGTTGTTCT-3′, VCAM-1 (reverse) 5′-CCCCTCATTCCTTACCACCC-3′. The primer sequences of human GAPDH, ICAM-1, Mid1 were as follows: GAPDH (forward) 5′-GGAGCGAGATCCCTCCAAAAT-3′, GAPDH (reverse) 5′-GCTGTTGTCATACTTCTCATGG-3′; ICAM-1 (forward) 5′-ATGCCCAGACATCTGTGTCC-3′, ICAM-1 (reverse) 5′-GGGGTCTCTATGCCCAACAA-3′; Mid1 (forward) 5′-CTGACCTGCCCTATTTGTCTG-3′, Mid1 (reverse) 5′-GCACAGTGTGATACTAGGATGC-3′. PCR amplifications were performed by Agilent AriaMx Real-Time PCR System (Agilent Technologies, Santa Clara, CA, USA) and analyzed with 2^–∆∆Ct^ method.

### 4.8. Flow Cytometry

For detection of ICAM-1 expression, eEnd.2 cells were transfected with silencer select negative control siRNA or Mid1 silencer select siRNA (Ambion), as described above, and then stimulated with 100 ng/mL TNF-α (PeproTech) for 3 h. Cells were washed twice with PBS, and single cell suspension was prepared by digestion with Accutase (A6964, Sigma-Aldrich). Samples were centrifuged (400× *g*, 5 min) and pellets were resuspended and fixed with 2% formaldehyde. After washing two times with PBS containing 2% FBS, cells were incubated with an anti-CD16/CD32 for 10 min to block FcϒIII/IIRs and then incubated with a fluorescein isothiocyanate (FITC)-conjugated anti-mouse ICAM-1 (YN1/1.7.4; Biolegend, London, UK) antibody for 20 min. Mean fluorescence intensity (MFI) of ICAM-1 expression was detected using Cytoflex flow cytometer (Becton Dickinson, Mountain View, CA, USA).

### 4.9. Adhesion Assay

For detection of neutrophil adhesion on stimulated eEnd.2 cells, cells were transfected and stimulated as described above. After stimulation, wells were washed twice with PBS, and 2 × 10^6^ freshly isolated neutrophils were added in each well and incubated, at 37 °C, for 1 h. Cells were washed with PBS twice, collected after digestion with Accutase (Thermofisher) and centrifuged for 10 min (15,300× *g*, 4 °C). Neutrophil adhesion to activated endothelial cells were measured in terms of MPO levels (ng/mL) in each well. Briefly, pellets were resuspended in 0.2 M PB buffer, pH7.4, then centrifuged, and the pellets were again suspended in 1 mL of 0.5% hexadecyl-trimethylammonium bromide buffer. The dissolved samples were frozen for 24 h, thawed, sonicated for 90 s and put in a water bath (60 °C, 2 h). MPO reaction with substrate (3,3′,5,5′-Tetramethylbenzidine, TMB) (Sigma Aldrich)) was measured with spectrophotometer at 450 nm web length, with a reference filter at 540 nm, at 25 °C.

### 4.10. Confocal Microscopy

Neutrophil adhesion and ICAM-1 expression on stimulated eEnd.2 cells were further confirmed by confocal microscopy. For immunofluorescence imaging of ICAM-1 and Ly6G-positive cells, eEnd.2 cells were grown to 70% confluence and then cells were transfected and stimulated on glass coverslips as described above. After stimulation, cover slips were washed twice with PBS, and 2 × 10^6^ freshly isolated neutrophils were added in each well and incubated, at 37 °C, for 1 h. Cells were washed with PBS twice and then fixed with 1% formaldehyde for 10 min and then washed two times with PBS containing 2% fetal bovine serum. Samples were then incubated with primary antibodies: fluorescein isothiocyanate (FITC) conjugated anti-ICAM-1 antibody (ab206633; Abcam, Cambridge, UK) and phycoerythrin (PE) conjugated anti-Ly6G antibody (ab12327; Abcam, Cambridge, UK) in PBS containing 2% BSA overnight. After immunostaining, coverslips were rinsed with PBS twice and then stained with Hoechst 33,258 (Thermo Scientific) for 10 min. ProLong Diamond Antifade Mountant (Thermo Scientific) was added on all coverslips before putting on the slides. Confocal z-stakes images were taken using LSM 800 confocal microscope (Carl Zeiss, Jena, Germany) and orthogonal projection images were created using all slices for a total height of ~10 μm. Images were taken by using a ×63 oil immersion objective (numeric aperture = 1.25) and processed later using ZEN2012 (Carl Zeiss, Germany) software.

### 4.11. Western Blot

eEnd.2 cells were seeded and grown to 70% confluency, then transfected and stimulated as described above. After 1 h stimulation, cells were lysed on ice by Pierce RIPA buffer (Thermo Fisher Scientific) with 1% protease inhibitor cocktail (Thermo Fisher Scientific) for 15 min. After centrifugation (14,000 rpm, 5 min), supernatant was collected and protein concentration was determined with Pierce bicinchoninic acid (BCA) protein assay kit (Thermo Fisher Scientific). Then, 10 μg of protein was loaded per lane and separated on 8–16% Mini-PROTEAN TGX Stain-free precast gels (Bio-Rad Laboratories, CA, USA). Bio-Rad CheMidocTM MP imaging system (Bio-Rad Laboratories, CA, USA) was used to image the total protein gel. Total protein was then transferred to polyvinylidene fluoride membranes (Bio-Rad Laboratories, CA, USA) and imaged by Bio-Rad CheMidocTM MP imaging system (Bio-Rad Laboratories). To block the unspecific bindings, the membranes were blocked in the TBS/Tween 20 buffer with 5% non-fat milk powder for 1 h at room temperature, then immunoblotted by mouse monoclonal anti-PP2A, C subunit 1:1000 (Sigma-Aldrich, St. Louis, MO, USA) antibody overnight at 4 °C. The membranes were then incubated with horse anti-mouse secondary antibody 1:5000 conjugated with horseradish peroxidase (Cell Signaling Technology, MA, USA), at room temperature, for 1 h. Protein bands were imaged by Bio-Rad ChemiDocTM MP imaging system Image Lab™ software version 6.1 was used to normalize target protein band signal against the total protein of respective lane before calculation.

### 4.12. Statistics

Data are presented as means ± SEM, and n indicates number of animals or experiments for each group. For Mid1 expression analysis in RNAseq data, DESeq2 method was utilized. For all other data, statistical comparisons between two groups were performed by Mann–Whitney rank sum test. *p* < 0.05 was considered significant. For dose–response experiment, multiple comparisons were performed using Kruskal–Wallis one-way ANOVA on ranks, followed by Dunn’s method. Statistical analyses were performed by using GraphPad Prism 8 software (GraphPad Software, La Jolla, CA, USA).

## Data Availability

All datasets analyzed or generated during the study are available on request to corresponding author. RNAseq data analyzed in this study are available at Gene Expression Omnibus (GEO) database of NCBI (National Center for Biotechnology Information) (GEO Accession: GSE169532).

## Supplementary Materials

The following supporting information can be downloaded at: https://www.mdpi.com/article/10.3390/ijms24010705/s1.
