# Supplementary material for: E3 Ubiquitin Ligase Midline 1 Regulates Endothelial Cell ICAM-1 Expression and Neutrophil Adhesion in Abdominal Sepsis
Source: Int J Mol Sci. 2022 Dec 31;24(1):705. doi: 10.3390/ijms24010705 (PMC9821100; doi:10.3390/ijms24010705)

Supplementary Figure S1

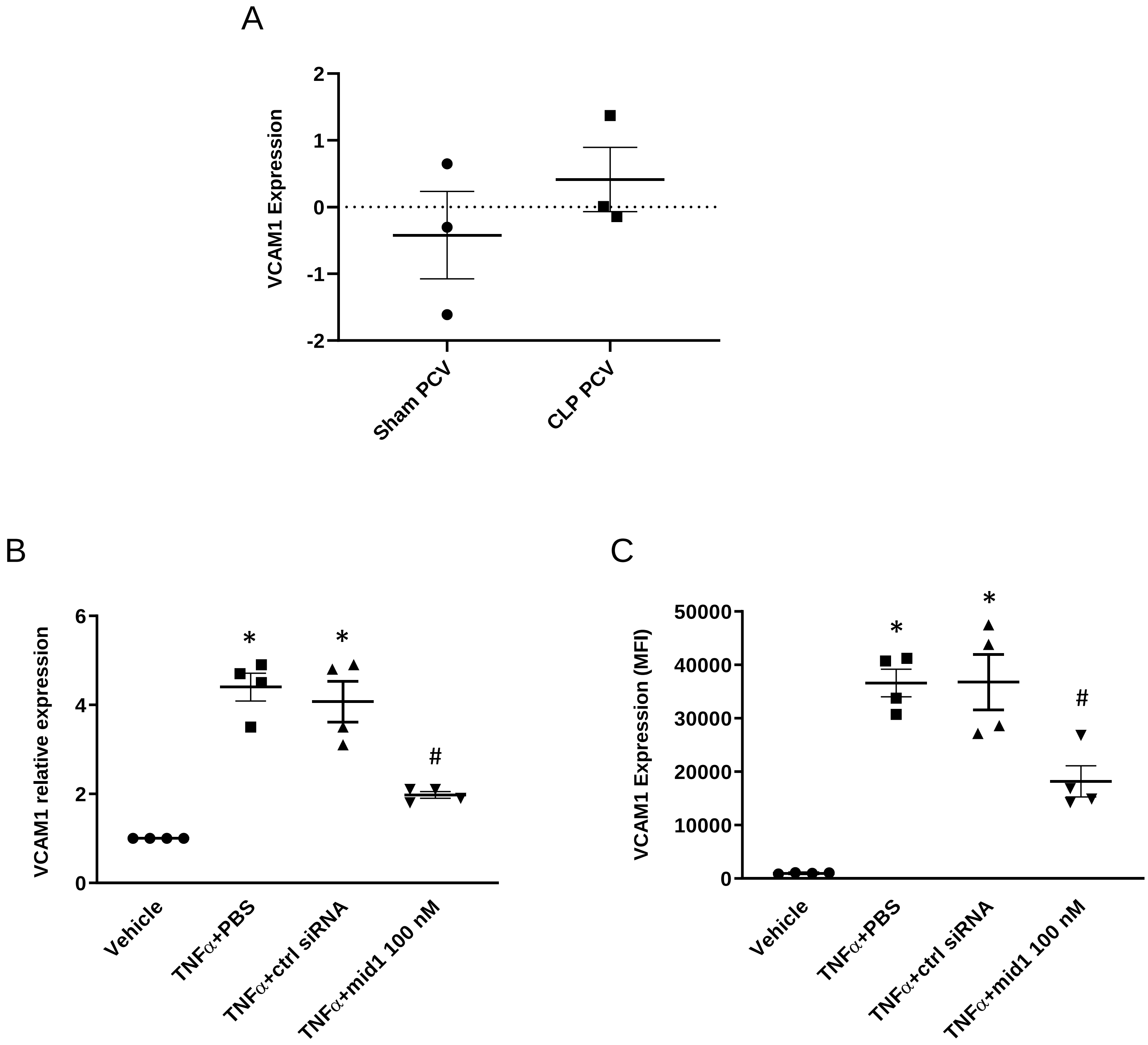

Supplementary Figure S1. VCAM-1 expression in lung post capillary venules of septic mice and endothelial cell lines. (A) VCAM-1 expression in the lung post capillary venules. Expression was analyzed in the RNAseq data using R program (DESeq2). Samples were collected 4h after induction of abdominal sepsis and sham mice served as negative control, n=3. (B, C) eEnd.2 cells were transfected with 100 nM negative control siRNA and Mid1 siRNA for 24h, then stimulated with 100 ng/ml TNF-α. (B) VCAM-1 mRNA expression was determined by RT-qPCR 1h after stimulation. (C) VCAM-1 surface expression was determined by flow cytometry 3h after stimulation. *p < 0.05 vs Vehicle, #p < 0.05 vs TNF-α + Control siRNA.

Supplementary Figure S2

Experiment 1

Western blot of Figure 4

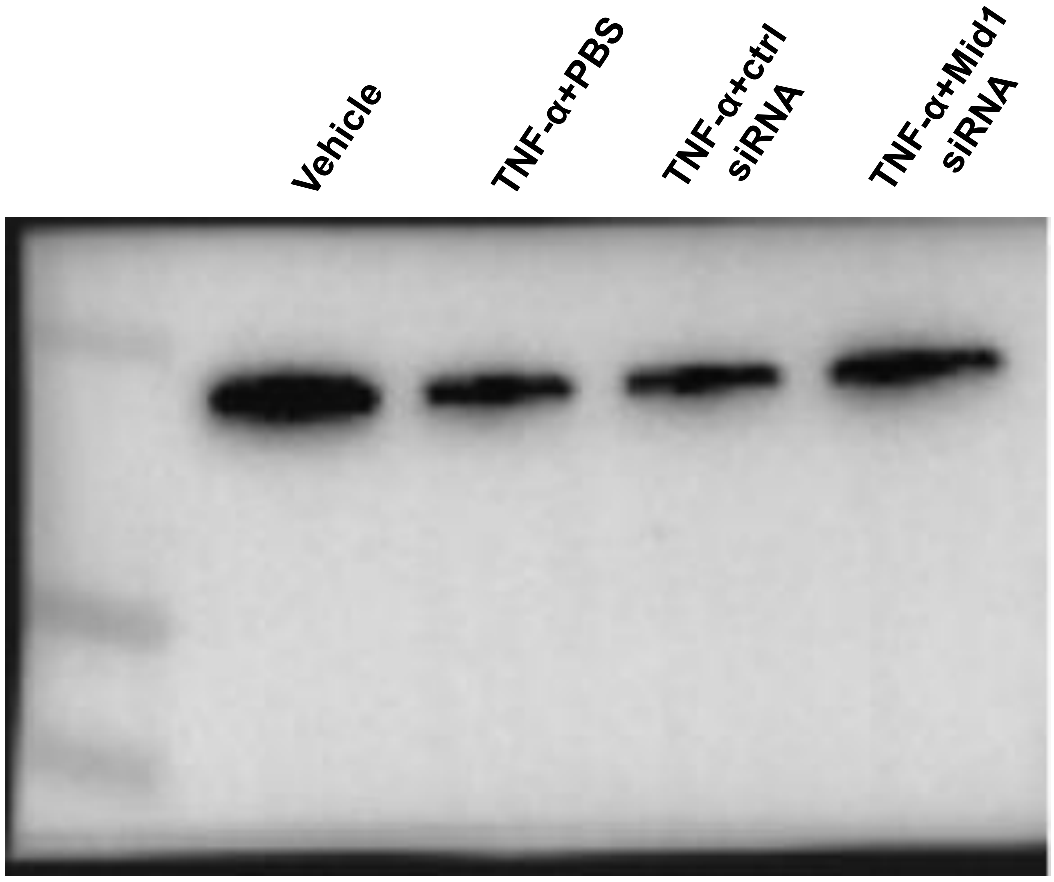

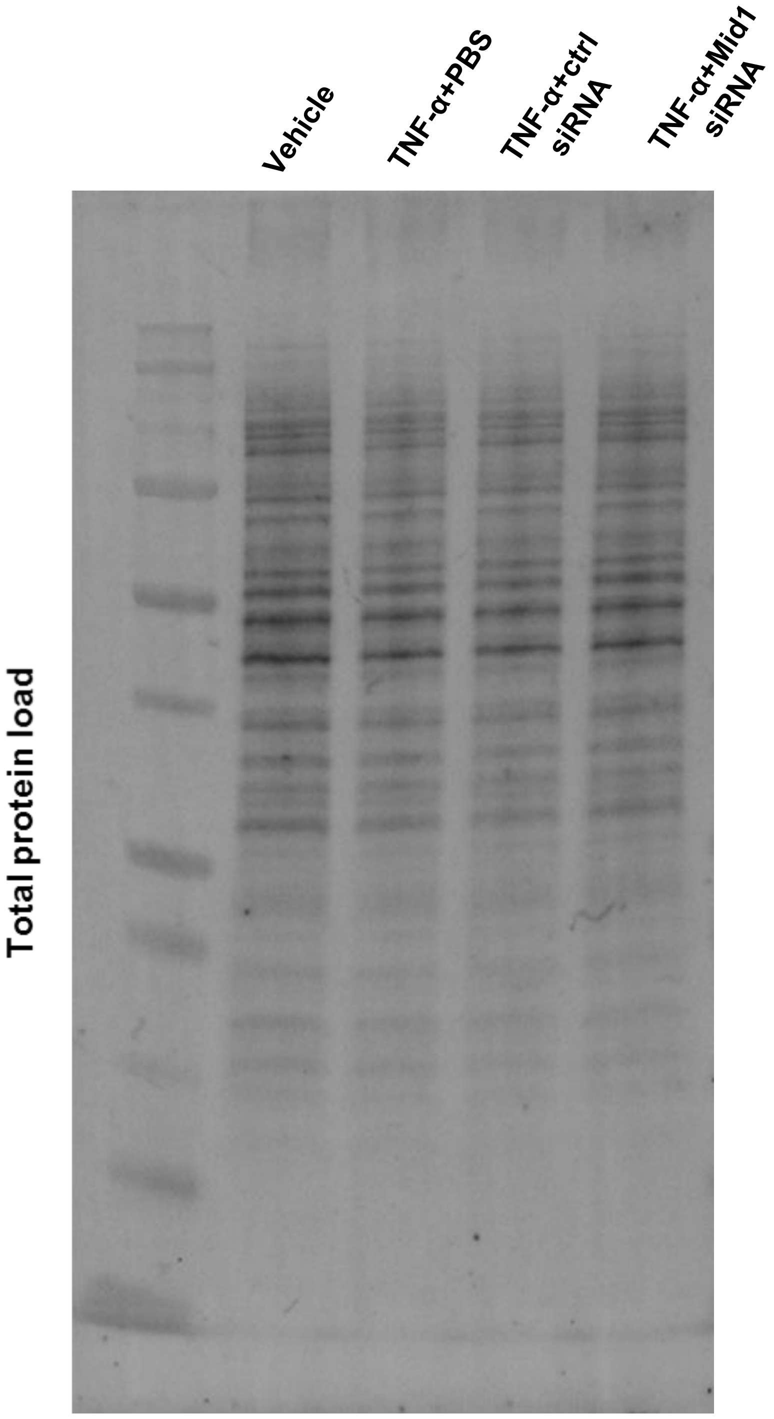

Supplementary Figure S2. Four (Experiment 1-4) original unadjusted western blot replicates of PP2Ac of figure 5. eEnd.2 cells were transfected with 100 nM negative control siRNA or Mid1 siRNA for 24 h, then stimulated with 100 ng/ml TNF-α for 1 hour. PP2Ac band intensity was normalized with total protein load of the respective lane and then relative band intensity was calculated by Bio-Rad ChemiDocTM MP imaging system Image Lab™ software described details in the method section.

Supplementary Figure S2

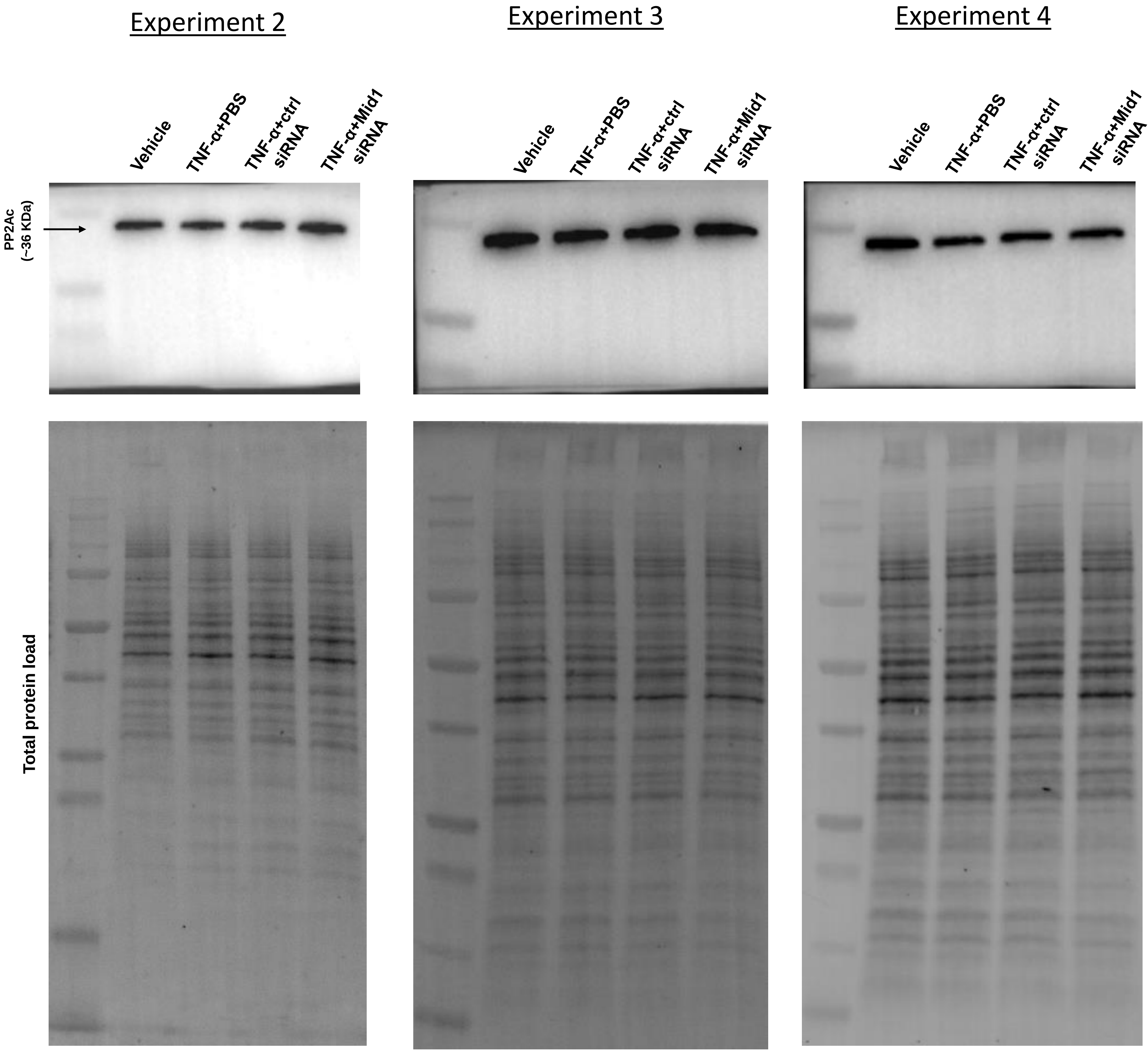

Supplement: Supplementary file 1 [file ijms-24-00705-s001.zip › ijms-1691351-supplementary.pdf]
